# Retinal Pigment Epithelium and Müller Progenitor Cell Interaction Increase Müller Progenitor Cell Expression of PDGFR*α* and Ability to Induce Proliferative Vitreoretinopathy in a Rabbit Model

**DOI:** 10.1155/2012/106486

**Published:** 2012-08-23

**Authors:** Gisela Velez, Alexa R. Weingarden, Budd A. Tucker, Hetian Lei, Andrius Kazlauskas, Michael J. Young

**Affiliations:** ^1^Department of Ophthalmology, University of Massachusetts Medical School, Worcester, MA 01605, USA; ^2^Department of Ophthalmology, Harvard Medical School, Boston, MA 02115, USA; ^3^The Schepens Eye Research Institute, Massachusetts Eye and Ear, Boston, MA 02114, USA

## Abstract

*Purpose*. Proliferative vitreoretinopathy (PVR) is a complication of retinal detachment characterized by redetachment of the retina as a result of membrane formation and contraction. A variety of retinal cells, including retinal pigment epithelial (RPE) and Müller glia, and growth factors may be responsible. Platelet-derived growth factor receptor alpha (PDGFR**α**) is found in large quantities in PVR membranes, and is intrinsic to the development of PVR in rabbit models. This study explores the expression of PDGFR in cocultures of RPE and Müller cells over time to examine how these two cell types may collaborate in the development of PVR. We also examine how changes in PDGFR**α** expression alter Müller cell pathogenicity. *Methods*. Human MIO-M1 Müller progenitor (MPC) and ARPE19 cells were studied in a transmembrane coculture system. Immunocytochemistry and Western blot were used to look at PDGFR**α**, PDGFR**β**, and GFAP expression. A transfected MPC line cell line expressing the PDGFR**α** (MIO-M1**α**) was generated, and tested in a rabbit model for its ability to induce PVR. *Results*. The expression of PDGFR**α** and PDGFR**β** was upregulated in MIO-M1 MPCs cocultured with ARPE19 cells; GFAP was slightly decreased. Increased expression of PDGFR**α** in the MIO-M1 cell line resulted in increased pathogenicity and enhanced ability to induce PVR in a rabbit model. *Conclusions*. Müller and RPE cell interaction can lead to upregulation of PDGFR**α** and increased Müller cell pathogenicity. Müller cells may play a more active role than previously thought in the development of PVR membranes, particularly when stimulated by an RPE-cell-rich environment. Additional studies of human samples and in animal models are warranted.

## 1. Introduction

Proliferative vitreoretinopathy (PVR) occurs in 5–10% of rhegmatogenous retinal detachments [1]. It is a complex cellular process characterized by preretinal and subretinal membrane formation, intraretinal degeneration, gliosis, and contraction. The disease is characterized by (1) migration and proliferation of retinal pigment epithelial cells (RPE) and glial cells along with synthesis of extracellular matrix (ECM) proteins, such as collagen or fibronectin, which organize into retinal and vitreous membranes; (2) intraretinal glial cell proliferation, photoreceptor degeneration, and disorganization of retinal cell layers [2, 3]. In a way, PVR can be viewed as maladaptive and/or aberrant wound healing [4], the severity of which is often determined by the circumstances in which it occurs.

Certain clinical characteristics are associated with an increased risk of PVR development [5, 6]. These can be classified into two categories. In the first group are risk factors which increase RPE cell dispersion into the subretinal and preretinal space, and cellular proliferation. These include large retinal tears and detachments, cryotherapy and sclera indentation, and retinal detachments of long duration. In the second group are characteristics which increase the presence of growth factors and inflammatory cytokines in the environment, with or without breakdown of the blood-ocular barrier [7]. This includes vitreous hemorrhage, choroidal hemorrhage, and cryotherapy. These risk factors for PVR are additive—the more characteristics, the higher the risk of PVR development. Patients with traits in both categories are faced with a perfect storm, in which cell migration and proliferation occur in an environment primed for cellular misbehavior.

There has been controversy in the literature regarding the extent of involvement of cells other than RPE, such as Müller glia, in the pathogenesis of PVR. It is a fact that Müller cells are active participants. Recent work demonstrating the reactivity of Müller glia during retinal detachment and other forms of retinal injury suggests that these cells play a significant role in diseases involving retinal injury and degeneration, such as PVR. Although RPE cells have long been considered the principal mediators of this disease, Müller cell activation, migration, proliferation and transformation in retinal detachment, and retinal injury have all been documented [8–10]. Increased expression of GFAP and vimentin, indicative of increased reactivity, has been demonstrated in Müller glia in detached human retinas and experimental models of retinal detachment [11, 12]. Experimental detachment models have also shown Müller cell proliferation which peaks at 3-4 days retinal detachment and continues at a slower rate for weeks to months [13], as well as migration of Müller cell processes and nuclei throughout the retinal layers and into the subretinal space [8]. Certainly, the data supports the need to explore more closely how these cells participate in PVR pathogenesis, and what drives them to do so.

The question then arises of whether the behavior of Müller cells, already primed and activated in the context of retinal detachment, can be altered by the presence of RPE cells and growth factors in the vitreous environment. Our experiments were designed to help us understand how RPE and Müller cells might affect each other when forced to interact in the context of retinal detachment, and how Müller glia altered by this environment participate in PVR. Because our goal is to better understand this process in human disease, we have chosen to work with the ARPE19 and MIO-M1 Müller progenitor cell lines.

## 2. Methods

### 2.1. Major Reagents

Antibodies against PDGFR*α* and PDGFR*β* were purchased from Cell Signaling Technology (Beverly, MA, USA), anti-GFAP from Zymed (San Francisco, CA, USA), and *β*-actin from Abcam (Cambridge, MA, USA). Secondary antibodies (antirabbit IgG) were purchased from Jackson ImmunoResearch Laboratories, Inc (West Grove, PA, USA). ARPE19 cells were purchased from American Type Culture Collection; MIO-M1 Müller progenitor cells (MIIO-M1 MPCs) were obtained by material transfer agreement from the Institute of Ophthalmology, University College London, from Drs. GA Limb and Professor PT Khaw (patent application PCT/GB2004/005101). Primary rabbit conjunctival fibroblasts (RCFs) were obtained as previously described [14].

### 2.2. Cell Cultures

Transmembrane cell cultures were set up using MIO-M1 and ARPE19 cells in DMEM/F12 media with 10%FBS (Gibco). MIO-M1 cells were plated in six-well plates and allowed to reach confluency. ARPE19 cells were plated on transwell inserts with 0.4 *μ*m pores and allowed to grow to confluency. Upon reaching confluency, the inserts containing ARPE19 cells were placed in MIIO-M1 containing six-well plates and cultured in a total volume of 3 mL of media. Control groups of each single-cell type were grown concurrently. Cells were fed with 1.5 mL of media on day 2 and 6, with complete media changes at day 4.

### 2.3. Western Blotting

MIO-M1 and ARPE19 cells were harvested at days 1, 3, 5, and 7. Lysates were made for Western blot analysis. Media was removed and cells were collected from experimental and control plates and inserts using sterile cell scrapers in PBS. Lysates of these cells were created by incubation for 30 min at 4°C in Ripa buffer 50 mM Tris HCl [pH 8.0], 0.1% SDS, 0.5% sodium deoxycholate, 1% NP-40, 150 mM NaCl, 2% protease inhibitor cocktail, 2% phosphatase inhibitor cocktail 1, and 2% phosphatase inhibitor cocktail 2 (Sigma), followed by 15 seconds of sonication and removal of cellular debris by centrifugation at 12,000 rpm for 12 min at 4°C. The protein content of the lysates was determined with the BCA protein assay (Thermo Scientific). Expression of PDGFR*α* and PDGFR*β* in the control and experimental samples was compared by Western blotting. 50 *μ*g of protein was resolved by 10% sodium dodecyl sulfate-polyacrylamide gel electrophoresis (SDS-PAGE). The protein bands were transferred onto nitrocellulose membranes (Bio-Rad Laboratories), and the membrane was subjected to Western blot analysis using anti-PDGFR*α* or anti-PDGFR*β* primary antibodies. Band density was quantified using Image J and normalized to *β*-actin expression.

### 2.4. Immunocytochemistry

MIO-M1 and ARPE19 cells were cocultured as described above on 16-well glass slides with ARPE19 cells in 8-well strips of 0.2 *μ*m membrane inserts. Inserts were removed after 5 days and the plated cells were fixed for 20 minutes with 4% paraformaldehyde and permeabilized for one hour at room temperature with 10% goat serum and 0.1% Triton X-100 in PBS. MIO-M1 cells were stained with primary antibodies directed against PDGFR*α*, PDGFR*β*, and incubated at 4°C overnight. Primary and secondary antibodies were prepared in 10% GS in PBS solutions at a concentration of 1 : 100 for PDGFR*α* and *β*. The slides were then washed with PBS. Secondary antibodies were added to the slides for 1 hour. Slides were then washed with PBS, coated with mounting media containing DAPI, and covered. The cells were examined by fluorescent microscopy with Cy2 filter.

### 2.5. Preparation of MIO-M1*α* Cell Line and Rabbit Model for PVR

The pLHDCX^2^-PDGFR*α* retrovirus was used to stably express the PDGFR*α* in immortalized MIO-M1 Müller progenitor cells (MIO-M1 MPCs). Transfected cells with increased PDGFR*α* expression were selected for resistance to histidinol toxicity and designated as MIO-M1*α*.

PVR was induced in the right eyes of pigmented rabbits purchased from Covance (Denver, PA, USA). Briefly, a gas vitrectomy was performed by injecting 0.1 mL of perfluoropropane (C_3_F_8_) (Alcon, Fort Worth, TX, USA) into the vitreous cavity 4 mm posterior to the corneal limbus. One week later, all rabbits received two injections: (1) 0.1 mL of PRP (platelet-rich plasma) and (2) 0.1 mL DMEM containing 2 × 10^5^ of rabbit conjunctival fibroblasts (RCFs), MIO-M1, and MIO-M1*α* cells. The extent of retinal detachment was evaluated by indirect ophthalmoscopy with a handheld +30 D fundus lens at days 2, 4, 7, and weekly thereafter for a total of 4 weeks. Extent of PVR was graded according to the Fastenberg classification from grade 0 through 5 [15]. On day 28 the animals were sacrificed and the eyes were enucleated. All surgeries were performed under aseptic conditions and pursuant to the ARVO Statement for the Use of Animals in Ophthalmic and Vision Research. The protocol for the use of animals was approved by the Schepens Animal Care and Use Committee. Mann-Whitney test for nonparametric data (*P* < 0.05) was used for statistical analysis.

## 3. Results

As previously discussed, the goal of these experiments is to determine if RPE cells induce a change in Muller cells that would result in a PVR-inducing cell.

**Figure 1:**
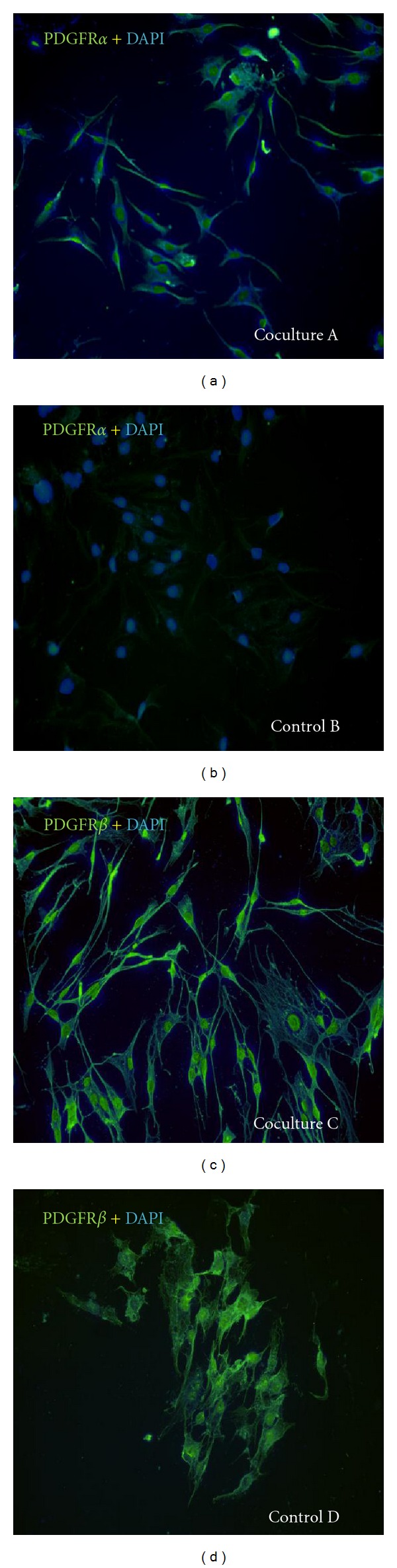
Immunocytochemical staining for PDGFR*α* and *β* (green) in MIO-M1 MPCs. Expression of PDGFR*α* after 5 days when cocultured with ARPE19 cells (A) and expression of PDGFR*α* in control culture (B). Expression of PDGFR*β* after 5 days when cocultured with ARPE19 cells (C) and expression of PDGFR*β* in control culture (D). PDGFR*β* expression appears relatively stable, while PDGFR*α* expression increases more significantly.

**Figure 2:**
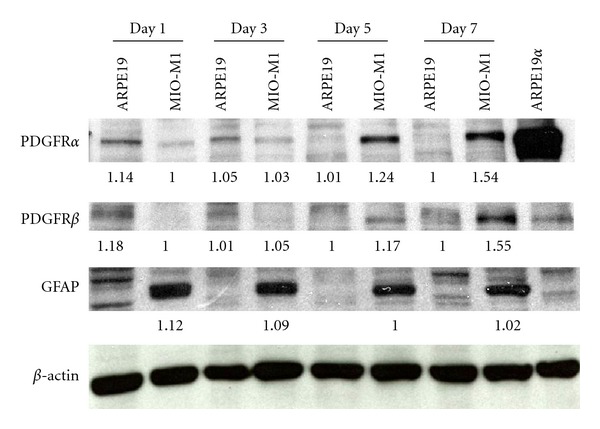
Western blot analysis of coculture samples at days 1, 3, 5, and 7 shows noticeable increases in PDGFR*α* expression in MIO-M1 MPCs. Transfected ARPE19 cells with increased expression of PDGFR*α* (RPE*α*) are used as control for comparison. PDGFR*β* levels remain relatively low in both cell types throughout the course of the experiment, with a small increase in MIO-M1 MPCs on day 5 and a more significant increase by day 7. MIO-M1 cells show a small but measurable and consistent decrease in expression of GFAP between days 1 and 7 of the experiment. RPE*α* cells are included for control. Protein concentrations were standardized by Bradford assay. Band intensity was standardized by *β*-Actin expression (shown). Relative expression ratios are shown.

Immunocytochemical staining of MIO-M1 MPCs after 5 days of coculture with ARPE19 cells confirms MIO-M1 MPCs upregulate their expression of PDGFR*α* (Figures 1(a)-1(b)), with a moderate increase in expression of PDGFR*β* (Figures 1(c)-1(d)). Consistent with these results, Western blot analysis showed upregulation of PDGFR*α* expression in experimental MIO-M1 MPCs by day 5 after-plating (Figure 2). In contrast, expression of PDGFR*β* remained low in MIO-M1 and ARPE 19 cells across the same time, with a measurable increase in MIO-M1 cells by day 7 only (Figure 2). A small but detectable decrease in GFAP expression was observed in MIIO-M1 MPCs as early as day 3 after coculture (Figure 2).

**Figure 3:**
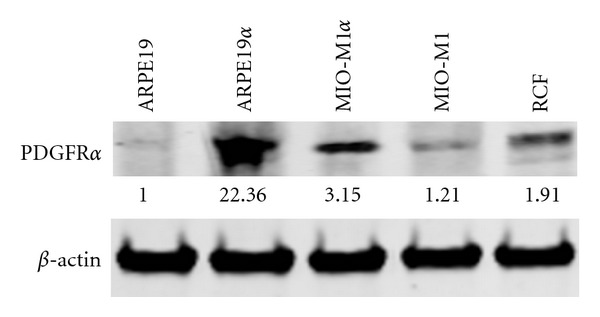
Western blot of ARPE19, ARPE19*α*, MIO-M1 MPCs, MIO-M1*α*-transfected MPCs, and rabbit conjunctival fibroblast (RCF) lysates shows comparable expression of PDGFR*α* in transfected MPCs (MIO-M1*α*) and RCFs, with levels comparable to those developed in cocultured cells. MIO-M1*α* MPCs have an approximate 2.56X increase in PGFR*α* expression when compared to untransfected MIO-M1 MPCs, and a 1.65X increase in expression when compared to RCFs. Relative expression ratios are shown.

**Figure 4:**
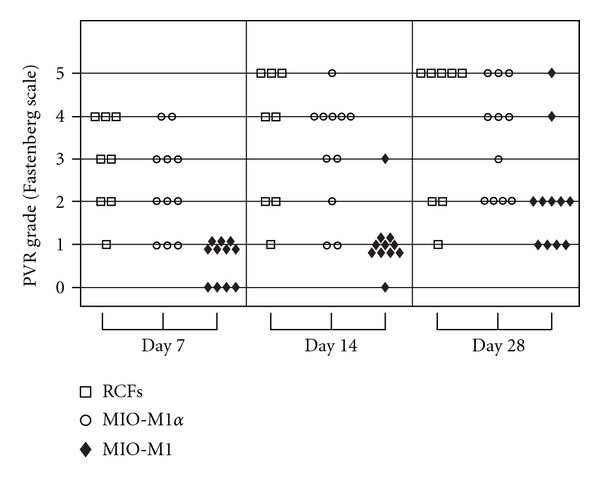
Overexpression of PDGFR*α* in MIO-M1 MPCs induces a phenotypic switch to fibroblast-like cells that effectively induce PVR postintravitreal injection in rabbit. Classification is as follows: stage 0—no PVR; stage 1—presence of fibrous bands; stage 2—fibrous bands with traction; stage 3—retinal detachment involving less than 2 quadrants; stage 4—retinal detachment involving more than 2 quadrants; stage 5—total retinal detachment. For statistical purposes, stage 3 or higher is considered severe PVR. Mann-Whitney statistical analysis for nonparametric data showed a statistically significant difference (*P* < 0.05) at all three time points between RCFs versus MIO-M1 MPCs, and MIO-M1 MPCs versus MIO-M1*α* MPCs. No statistically significant difference was observed between RFCs versus MIO-M1*α* MPCs.

The above data suggests that upregulation of PDGFR*α* could lead to stimulation of fibroblastic behavior consistent with PVR in Müller cells. Given our knowledge of the importance of PDGFR*α* and not PDGFR*β* (see discussion below), we overexpressed PDGFR*α* in MIO-M1 MPCs to test our hypothesis that Müller cells alone can induce PVR. As confirmed by Western blot analysis, transfection of MIO-M1 cells with pLHDCX^2^-PDGFR*α* resulted in increased expression of PDGFR*α* (Figure 3) in MIO-M1 cells comparable to that observed in cells cocultured with ARPE19 and with the expression of rabbit conjunctival fibroblasts (RCFs). Comparison of MIO-M1, MIIO-M1*α*, and RCF behavior in a rabbit model showed a dramatic increase in PVR pathogenicity of the MIIO-M1*α* cell line comparable to that of RCF's. MIIO-M1*α* cells were equally effective as RCFs in inducing PVR after-transplantation (Figure 4).

## 4. Discussion

PDGFR*α* has long been implicated in the pathogenesis of PVR. It is found extensively in preretinal membranes from PVR patients [16, 17]. Experimental models using mouse embryonic fibroblasts as well as rabbit conjunctival fibroblasts have demonstrated the intrinsic role that PDGFR*α*, and not PDGFR*β*, plays in the pathogenesis of the disease [14, 18]. In fact, inhibition of the PDGFR*α*, either through inhibition of its tyrosine kinase or the ROS pathway, has been shown to be sufficient in these models to attenuate and/or inhibit the development of PVR [19, 20].

Studies show that Müller cells are present in PVR membranes [21]. However, RPE rather than Müller cells, have dominated the literature and have been the focus of most studies and theories of PVR [22, 23]. This is partly due to the fact that RPE cell markers are more abundant in PVR membranes. The assumption has been made that Müller cells play a less important role, yet this may not be the case.

Our in-vitro study observations and correlating rabbit model results suggest that one of the mechanisms by which Müller cells may play a role in PVR is by upregulating the expression of PDGFR*α*. When comparing with previous studies in the literature, this change has a bigger impact on Müller cell behavior than RPE cell behavior [24]. These experiments also suggest that Müller cell up-regulation of PDGFR might be the result of the changes in the RPE cell rich environment which exists in retinal detachments with high-risk characteristics. High-risk retinal detachments for the development of PVR are more commonly characterized by increased RPE cell migration and presence in the vitreous cavity. In the presence of RPE cells in our study, Müller progenitor cells do two very important things—they change their expression of traditional Müller cell markers such as GFAP, and they increase their expression of PDGFR. Though these changes do not appear to be significant at first glance, studies in the rabbit model suggest that only a small increase in PDGFR*α* expression is necessary to dramatically alter the behavior of these cells to resemble that of fibroblasts. This is in stark contrast to studies performed using RPE cells, in which a more than 80-fold increase in receptor expression was necessary to significantly alter their behavior, with invivo results which were still inferior when compared to those using fibroblasts [24].

Müller cell plasticity and their capacity to transform their phenotype have already been demonstrated [25, 26]. The ability of this cell type to alter its expression of GFAP in different environments helps to support our observations [9]. Dedifferentiation in particular is thought to play an important role in how Müller cells participate in PVR. Müller cells in the peripheral retina, where PVR most often occurs, have been shown to express stem cell markers, indicative of active proliferation, and dedifferentiation [27]. In-vitro, human-derived Müller cells, including the MIO-M1 Müller progenitor cell line, have been shown to exhibit neural stem cell traits [28, 29].

We therefore propose the following theory to PVR development invivo. During retinal detachment, depending on the size and longevity of the detachment as well as the size and location of the retinal tear, there is an opportunity for RPE cells to abandon their natural monolayer and migrate onto the subretinal and preretinal space. The vitreous often acts as a scaffold onto which these cells can attach. Once allowed to migrate, RPE cells begin to produce cytokines and cofactors (yet to be fully identified) which can alter Müller cell phenotype and growth factor surface protein expression, leading to an increase in fibroblastic behavior and pathogenicity. Whether there is complete transformation of Müller cells invivo remains a question. It is possible that the abundance of RPE cells in PVR membranes is a “red-herring”, and that RPE cells play more of an effector role, with Müller cells doing most of the membrane formation and contraction.

The drawback of our study is that it is based on observations in an in-vitro setup and a limited rabbit animal model. These observations would be more difficult to make invivo, given the progressive and dynamic nature of the disease and the plasticity of Müller cells. Despite its limitations, our in-vitro model allows us to capture specific changes, and our rabbit model allows us to confirm their importance.

Certainly more work needs to be performed invivo. The next step would be a series of experiments in retinal detachment models looking at intraretinal GFAP and PDGFR*α* expression. Another set of experiments would be aimed at identifying those molecules which trigger Müller cell transformation and growth factor receptor expression. Once identified, therapeutic interventions can be designed to interfere with and redirect this process.
